# Mycosis fungoides: is it a Borrelia burgdorferi-associated disease?

**DOI:** 10.1038/sj.bjc.6602997

**Published:** 2006-02-21

**Authors:** S Miertusova Tothova, S Bonin, G Trevisan, G Stanta

**Affiliations:** 1 ICGEB – International Centre for Genetic Engineering and Biotechnology, 99 Padriciano, Trieste 34012, Italy; 2 Department of Clinical, Morphological and Technological Sciences, University of Trieste, 1-Dermatology Unit, 2-Pathology Unit, Cattinara Hospital, 447 Strada di Fiume, Trieste 34149, Italy

**Keywords:** Borrelia burgdorferi, cutaneous T-cell lymphoma, aetiology, lyme disease, mycosis fungoides, polymerase chain reaction

## Abstract

Mycosis fungoides (MF) is the most frequently found cutaneous T-cell lymphoma with an unknown aetiology. Several aetiopathogenetic mechanisms have been postulated, including persistent viral or bacterial infections. We looked for evidence of Borrelia burgdorferi (Bb), the aetiologic agent of Lyme disease (LD), in a case study of MF patients from Northeastern Italy, an area with endemic LD. Polymerase chain reaction for the flagellin gene of Bb was used to study formalin-fixed paraffin-embedded lesional skin biopsies from 83 patients with MF and 83 sex- and age-matched healthy controls with homolocalised cutaneous nevi. Borrelia burgdorferi-specific sequence was detected in 15 out of 83 skin samples of patients with MF (18.1%), but in none out of 83 matched healthy controls (*P*<0.0001). The Bb positivity rates detected in this study support a possible role for Bb in the aetiopathogenesis of MF in a population endemic for LD.

Mycosis fungoides (MF) is a relatively rare non-Hodgkin's lymphoma arising from extranodal tissue. It is the most common type of cutaneous T-cell lymphoma (CTCL), characterised by a typically slow evolution and protracted course. Although previous epidemiological studies had shown its increasing incidence (Weinstock and Horm, 1988), this could be an artefact caused by improved diagnostic techniques (Morales Suarez-Varela *et al*, 2000). Despite a number of studies, the aetiology of this disease still remains to be determined. Postulated mechanisms include the persistence of viral or bacterial agents that could exacerbate and/or stimulate chronic T-cell clonal expansion and cutaneous inflammation (Siegel *et al*, 2000; Fierro *et al*, 2001).

Borrelia burgdorferi (Bb) is the aetiologic agent of Lyme disease (LD), the most common tick-transmitted disease in the northern hemisphere (Jaenson, 1991). It is a multisystem inflammatory disorder, which affects skin, nervous system, cardiovascular system, joints, muscles and eyes (Scarpa *et al*, 1994). About 80% of all LD cases in Europe present cutaneous symptoms (dermatoborrelioses) (Mullegger, 2004). Erythema migrans, borrelial lymphocytoma and acrodermatitis chronica atrophicans are three characteristic dermatoborrelioses, occurring in different clinical stages of the disease, but borrelial isolation has also been reported from lesional tissues of various cutaneous disorders (Mullegger, 2004).

The aim of the present study was to evaluate the role of Bb in pathogenesis of MF in the LD-endemic region of Friuli Venezia Giulia (FVG) in Northeastern Italy (Ciceroni and Ciarrocchi, 1998). We analysed skin biopsies of MF patients for the presence of the specific Bb genome using a highly sensitive polymerase chain reaction (PCR) method (Lebech, 2002).

## MATERIALS AND METHODS

This study included 83 patients with a clinical diagnosis of MF, who were diagnosed between 1993 and 2003 at the Dermatology Unit of The University of Trieste in Italy. Clinical data analysed in this study included age, sex, stage of disease, extracutaneous tumour involvement and disease duration at the moment of the histological diagnosis. The study was conducted according to the Declaration of Helsinki protocols. Lesional tissue biopsies were reviewed by two dermatopathologists (GG and GS), and classified according to the criteria of the WHO classification of malignant lymphoma (Jaffe, 2001) after precise immunohistochemical evaluation. In all cases, the diagnosis of MF was established by clinico-histopathological correlation between dermatologist GT and dermatopathologists GG and GT. Immunostaining was performed on paraffin-embedded tissue sections in all 83 MF cases using monoclonal antibodies specific for T-cell-associated antigens (CD3, CD4 and CD8) and for B-cell-associated antigens (CD20) and plasma cells.

Formalin-fixed and paraffin-embedded tissues of skin biopsies from 83 MF patients were examined for the presence of Bb genome. As controls we used surgical excisions of homolocalised naevi with the surrounding skin (63 dermal nevi, eight junctional nevi, six compound nevi and six blue nevi) of 83 healthy subjects, matched for age, sex and skin location.

Total DNA was extracted from 10 *μ*m sections of paraffin-embedded tissue blocks using a phenol–chloroform method (Pauluzzi *et al*, 2004). We avoided a nested PCR because of the high risk of carryover related to this method. As the DNA extracted from formalin-fixed and paraffin-embedded tissues is partially degraded, we decreased the amplicon size to 75 bases and increased a number of amplification cycles to 70 in order to improve the sensitivity of the method (a high number of PCR cycles is suggested for paraffin-embedded tissue analysis) (Lehmann and Kreipe, 2001; Bonin *et al*, 2003). Primer sets were designed in low variability regions of the Borrelia chromosome. The primers targeted the gene that encodes flagellin, a 41 kDa protein (GenBank X16833) that is conserved in all European species of *Borrelia burgdorferi sensu lato* (Schwaiger *et al*, 2001). Amplification employed bp 775–793 as the forward primer and bp 849–829 as the reverse primer. The sensitivity and specificity of the primers has previously been tested (Pauluzzi *et al*, 2004). Every PCR reaction was run in duplicate under previously reported conditions (Pauluzzi *et al*, 2004).

Pure genomic Bb DNA from *Borrelia afzelii, Borrelia garinii* and *Borrelia sensu stricto* and Bb positive tissues were used as positive controls. Formalin-fixed and paraffin-embedded biopsies of acrodermatitis chronica atrophicans and erythema migrans were used as tissue positive controls. As negative controls, we used paraffin blocks without tissue and Bb negative tissues, which were skin lesions positive for Mycobacterium tuberculosis. In the set-up of the method to assess its specificity, we analysed scrapes of primary syphilis, the DNA from *Mycobacetrium avium* and *Candida albicans*, as previously reported (Pauluzzi *et al*, 2004). As additional critical controls, we have included 36 paraffin-embedded biopsies of B-cell pseudolymphomas.

Polymerase chain reaction products were confirmed by hybridisation with a ^32^P-labelled probe bp 801–828 internal to the amplicon, detected and counted using a Cyclon instrument (Packard) (Pauluzzi *et al*, 2004).

In Bb positive cases a molecular analysis for the rearrangement of T-cell receptor-gamma (TCR-*γ*) gene was performed. T-cell receptor-gamma monoclonality was analysed using a PCR technique as described by McCarthy *et al* (1992) and modified by Department of Dermatology, Medical University of Graz, Graz, Austria (Massone *et al*, 2005). Polymerase chain reaction products were concentrated by precipitation and then resupended in 20 *μ*l of TE buffer 1 × . A 10 *μ*l aliquot of the PCR product was run on a 3.5% Metaphor agarose gel, stained with ethidium bromide and viewed under the UV light (Massone *et al*, 2005). T-cell receptor-gamma gene rearrangements were considered monoclonal when one or two bands were produced within the expected size ranges (75–110 bases) (McCarthy *et al*, 1992). As positive controls we used DNA extracted from Jurkat cells and DNA extracted from non-Hodgkin lymph nodes where a T-cell monoclonality has been previously assessed.

Statistical analysis was performed with dedicated STATASE8 software (Stata Corporation, TX, USA). The statistical significance was evaluated by Fisher's exact test for a 2 × 2 contingency table (95% confidence intervals) and one-way analysis of variance (ANOVA) test. Kruskal–Wallis test was applied to compare age at diagnosis, sex and disease duration in MF patients positive and negative for Bb genome. Moreover, to estimate the joint effects of the above-mentioned covariates on disease duration, the data were analysed by fitting the Cox proportional hazards regression model. *P*-values of 0.05 or less were considered to indicate statistical significance.

## RESULTS

**Figure 1:**
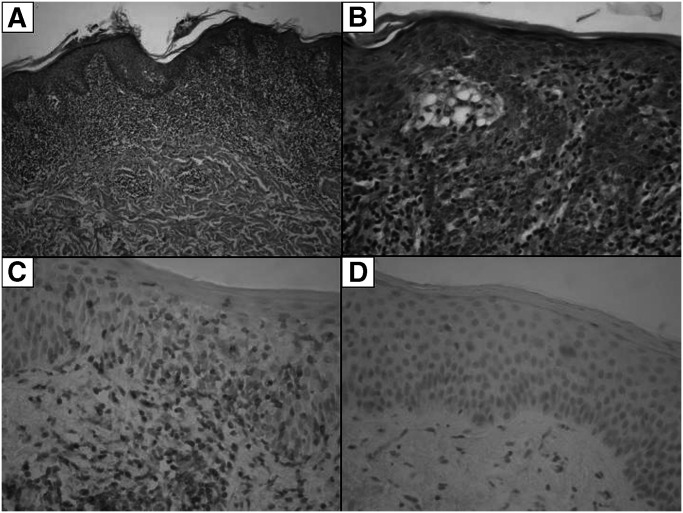
(**A**) The most common histological finding in our MF cases: a bandlike upper dermal infiltrate of lymphocytes, fibrosis of papillary dermis and the infiltration of lymphocytes into the epidermis (epidermotropism). (**B**) A detail of Pautrier's microabscesses. (**C**) CD4 positive immunohistochemistry. (**D**) CD20 negative immunohistochemistry.

In total, 83 MF patients were enrolled in the study, and patient characteristics are shown in Table 1. Skin biopsies were taken from MF lesions localised in five cases on the head and neck, in 43 cases on the trunk, in nine cases on the upper extremities, in 14 cases on lower extremities, and 12 cases were of unspecified localisation. The majority of our patients (87%) presented with early-stage disease (IA–IIA). In total, 13% of our patients had late-stage disease (IIB–IVB). Duration of the disease varied from 6 months to 15 years, mean 3.5 years. Immunohistochemistry: all cases examined were uniformly positive for CD3 and CD4 antigens and uniformly negative for CD20 and plasma cell antigens, 75% of samples were also CD8 positive (Figure 1).

The most common histological finding in our cases consisted of a bandlike upper dermal infiltrate of lymphocytes, fibrosis of papillary dermis and the infiltration of lymphocytes into the epidermis (epidermotropism) in the form of either a single lymphocyte epidermotropism or Pautrier's microabscesses. Lymphocytes infiltrating epidermis showed atypical features such as hyperchromatic and convoluted or cerebriform nuclei (Figure 1).

Using a sensitive PCR method with specific primers confirmed by hybridisation with a ^32^P-labelled Borrelia flagellin gene region oligonucleotide probe, Bb-specific sequence was detected in 15 out of 83 lesional skin samples of patients with MF (18.1%), but in none out of 83 matched healthy controls. The difference in frequency was significant (*P*<0.0001). In addition, six out of 36 of B-cell pseudolymphoma controls also tested positive for Bb genome analysis.

All the 15 MF samples positive for Bb genome were from patients that had early-stage disease. Seven out of 15 patients presented with stage IA disease, six had stage IB and two patients had stage IIA. The difference in stage between the group of Bb negative and Bb positive MF samples was not significant (*P*=0.99). No significant differences were detected between Bb positive and negative patients regarding age (*P*=0.313), sex (*P*=0.160) and disease duration (*P*=0.590). In the multivariate analysis, the analysed covariates (age, sex and Bb positive or negative results) did not have any effect on disease duration *P*=0.596).

**Figure 2:**
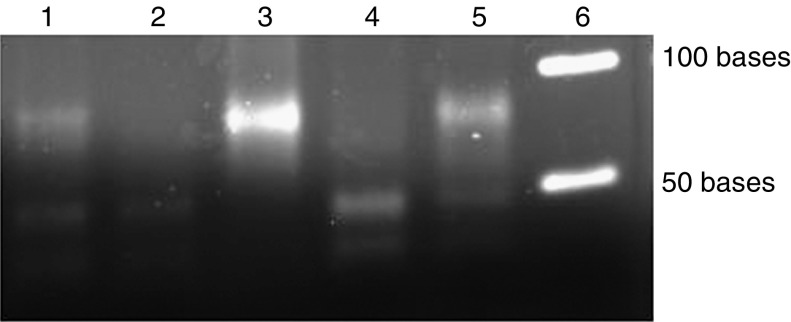
Examples of polymerase-chain reaction analysis of T-cell receptor-*γ* gene rearrangements. Lanes 1, 3 and 5 are MF samples positive for the rearrangements; lanes 2 and 4 are MF cases negative for the rearrangements. Lane 6 is molecular marker ladder.

In cases positive for the Bb genome, we analysed the TCR-*γ* gene rearrangements using PCR analysis. A monoclonal T-cell infiltrate was demonstrated in 12 out of 15 Bb positive cases (80%). In 10 cases, one band in the range of 80–85 bases was observed and in two cases two bands in the same range were found representing a biallelic rearrangement of the TCR-*γ* genes (Figure 2) (McCarthy *et al*, 1992).

## DISCUSSION

We have demonstrated the presence of Bb-specific DNA within lesional skin biopsies of MF patients, implying that approximately 18% of MF cases in this geographic area are perhaps related to infection with this organism. Despite LD being endemic in this region of FVG in Northeastern Italy (Ciceroni and Ciarrocchi, 1998), we have shown that this association is highly significant, since Bb DNA was not found in any of our healthy control subjects.

To confirm the diagnosis of MF we analysed the TCR-*γ* gene rearrangements using PCR analysis. The finding of identical (clonal) TCR-*γ* gene rearrangements in cutaneous T lymphocytes indicates a malignant proliferation and differs them from a non-clonal (reactive) T-cell infiltration. Thus, detection of clonal TCR-*γ* gene rearrangements by PCR is a valuable tool for the diagnosis of cutaneous or other T-cell lymphomas. Out of 15 Bb positive cases, 12 were found to be positive for the clonal rearrangements of TCR-*γ* chain gene. The percentage of positive cases (80%) for the TCR-*γ* gene rearrangement agrees with other studies (Klemke *et al*, 2002; Ponti *et al*, 2005). Negative cases may represent T-cell neoplasms with rearrangement not in the gamma chain locus but rather in the V-II family locus (McCarthy *et al*, 1992).

The diagnosis of MF is dependent on confirmatory tissue biopsy showing atypical skin-homing (epidermotropic) malignant T-helper memory phenotype (CD3+, CD4+, CD8−, CD45R0+) (Fierro *et al*, 2001). MF is hypothesised to arise through a persistent antigenic stimulation, leading to an accumulation of skin-homing T-cells that are defective in Fas-mediated apoptotic programmed cell death (Tan *et al*, 1974; Nagasawa *et al*, 2004).

Although viruses are known to provide chronic immune stimulation, the findings of their association with MF are controversial (Pancake *et al*, 1995; Boni *et al*, 1996; Nagore *et al*, 2000; Herne *et al*, 2003). HTLV-1 is found in adult T-cell lymphoma and leukemia, and it has been implicated by several studies in CTCL (Zucker-Franklin and Pancake, 1994; Pancake *et al*, 1995; Khan *et al*, 1996). However, others have not been able to confirm these findings (Boni *et al*, 1996; Wood *et al*, 1996). Epstein–Barr virus infection has also been previously implicated in several kinds of lymphomas, including peripheral T-cell lymphomas (Shimakage *et al*, 2001; Kanegane *et al*, 2002). Another study showed significantly higher CMV seropositivity rates in early-stage MF patients with normal immune systems and minimal skin involvement compared with control subjects (Herne *et al*, 2003). Latent CMV and/or EBV infection could provide chronic antigen stimulation, induce T-cell proliferation, and adversely affect the apoptosis of skin-homing memory/helper T-cells.

It has also been suggested that bacterial agents, such as *Staphylococcus aureus* (Tokura *et al*, 1992; Jackow *et al*, 1997) or *Chlamydia pneumoniae* (Abrams *et al*, 2001), might contribute to MF pathogenesis by acting as persistent antigens, thus exacerbating and/or perpetuating chronic T-cell expansion and cutaneous inflammation.

In Europe LD is caused by at least three species: *Borrelia burgdorferi sensu stricto, Borrelia afzelii* and *Borrelia garinii* (Wilske, 2003). Among the dominant genospecies of Bb isolated in the FVG region is Borrelia afzelii, with its known tropism for the skin (Ciceroni *et al*, 2001). The predominance of Borrelia afzelii in cutaneous lesions in LD has been already reported in several European countries (Rijpkema *et al*, 1997; Robertson *et al*, 1999; Ruzic-Sabljic *et al*, 2000; Ornstein *et al*, 2001). Strains of this intracellular pathogen can survive the adaptive immune response and persist in the skin despite a strong host antibody response (Pachner *et al*, 2004). It has been postulated that borrelia interacts with the complement, inactivating complement regulatory proteins (Kraiczy *et al*, 2001, 2002). Others have proved that Bb can hide in immunoprivileged sites (de Koning *et al*, 1995; Girschick *et al*, 1996). The antigen variation of the Bb outer membrane has also been discussed as a possible strategy for evading the immune response (Seiler and Weis, 1996; Zhang *et al*, 1997). Data from Perticarari *et al* (2003) suggest that spirochaetes, including Bb, are able to induce apoptosis in lymphocytes, and that the cells involved are prevalently CD4.

The immune response in humans with LD is characterised by a type 1-like cytokine response with the production of gamma interferon (IFN-*γ*), but no interleukin (IL)-4 (Forsberg *et al*, 1995; Oksi *et al*, 1996). The type 1 (Th-1) immune response with high production of IFN-*γ* has been suggested to be the optimal response to all infections caused by intracellular microbes, such as Bb. It stimulates phagocytosis, the intracellular killing of microbes, antigen presentation to T cells and secretion of pro-inflammatory cytokines (Shtrichman and Samuel, 2001). By clearing the pathogen, the Th-1 immune response would diminish further antigenic stimulation and allow a switch to a type 2 response by up-regulation of IL-4 (Spellberg and Edwards, 2001). However, if the Th-1 response fails to completely clear the infection, a persistent antigenic stimulation might induce chronic Th-1 immune responses with IFN-*γ* production. Data from Widhe *et al* suggest that an initial Borrelia- specific IFN-*γ* response, followed by up-regulation of IL-4, is associated with non-chronic manifestations of LD, whereas a persistent IFN-*γ* response may lead to chronic LD (Widhe *et al*, 2004).

Cutaneous lesions of MF are characterised by an epidermal Th1-type cytokine profile consisting of interleukin-2 and IFN-*γ*, whereas a type 2 cytokine production profile, consisting of IL-4, is more likely to occur in Sézary syndrome, the erythrodermic variant of MF (Saed *et al*, 1994). Human IFN-*γ* -inducible protein 10 (IP-10) is secreted by IFN-*γ* -stimulated keratinocytes (Sarris *et al*, 1997). It is chemotactic for CD4+ lymphocytes, a major component of MF lesions, and it probably accounts for the epidermotropism of CTCL (Sarris *et al*, 1995).

The inflammatory infiltrate in both the early and late skin manifestations of Lyme borreliosis is mainly composed of CD68+ macrophages and CD45RO+ memory T cells, with a predominance of CD4+ helper T cells (Silberer *et al*, 2000). Epidemiological data from many countries show that LD has increased significantly in incidence since the 1980s (Jaenson, 1991; Strle, 1999). The age-adjusted incidence rates of MF in our study area of 1 200 000 inhabitants were stable over the period of 6 years from 1995 to 2000, with the mean annual incidence of 1.9 cases per 100 000 person-years for males and 1.3 cases for females (Zanier, 1995–2000).

Given these findings, we suggest that Bb could play a cofactor role in the aetiology of a proportion of CTCL, of which MF is the most common type. Recognition that a proportion of CTCL is related to Bb infection may also have important therapeutic implications. If a causal link between some cases of MF and Bb infection could be confirmed, a specific antibiotic therapy might be useful to improve the disease outcome even though MF is a T-cell lymphoma. In fact, there are already reports of primary cutaneous B-cell lymphomas responding to antibiotic therapy designed to treat Bb infection (Hofbauer *et al*, 2001) even though the findings about the association between Bb and B-cell lymphomas are controversial (Goodlad *et al*, 2000; Kodama *et al*, 2005).

Another example of a tumour associated with chronic bacterial infection is gastric MALT (mucosa-associated lymphoid tissue) lymphoma, whose increased risk is clearly associated with *Helicobacter* (H) *pylori* infection, and which is the most frequent extranodal non-Hodgkin's lymphoma (Van Krieken and Hoeve, 2000). *H. pylori* can induce antigen-specific T-cell responses at the gastric site of infection and, in some cases, drives a long-lasting polarised Th1 response with the development of specific T cells leading to the onset of low-grade gastric MALT lymphoma (D'Elios *et al*, 2003). The identification and eradication of *H. pylori* causes prolonged remission in more than 70% of patients with MALT lymphoma (Boot and de Jong, 2002).

In conclusion, we have provided significant evidence to support the concept of antigen-driven lymphomagenesis in CTCL in response to Bb infection. We could hypothesise that in some individuals Bb might induce an antigen-specific long-lasting Th1 response leading to the accumulation of cutaneous CD4+ lymphocytes and their possible neoplastic transformation. A further assessment of the true incidence of Bb-associated CTCL is needed, together with further studies to assess the efficacy of antimicrobial therapy in treating this malignancy.

## Figures and Tables

**Table 1:** Characteristics of patients with mycosis fungoides

*Patients' data*	
Number of MF samples	83
Average age (years); range	65.5; 32–91
Median age (years); 25–75th percentile	66; 58–75
Sex male/female	49/34
	
*Stage*	
IA	33 (40%)
IB	28 (34%)
IIA	11 (13%)
IIB	4 (5%)
III	5 (6%)
IVA	0
IVB	2 (2%)
	
*Immunohistochemistry*	
CD3+	83/83
CD4+	83/83
CD8+	62/83
CD20−	0/83
Plasma cells	0/83

## References

[bib1] Abrams JT, Balin BJ, Vonderheid EC (2001) Association between Sezary T cell-activating factor, Chlamydia pneumoniae, and cutaneous T cell lymphoma. Ann NY Acad Sci 941: 69–85 11594584 10.1111/j.1749-6632.2001.tb03712.x

[bib2] Boni R, Davis-Daneshfar A, Burg G, Fuchs D, Wood GS (1996) No detection of HTLV-I proviral DNA in lesional skin biopsies from Swiss and German patients with cutaneous T-cell lymphoma. Br J Dermatol 134: 282–284 8746342

[bib3] Bonin S, Petrera F, Niccolini B, Stanta G (2003) PCR analysis in archival postmortem tissues. Mol Pathol 56: 184–186 12782767 10.1136/mp.56.3.184 PMC1187316

[bib4] Boot H, de Jong D (2002) Gastric lymphoma: the revolution of the past decade. Scand J Gastroenterol Suppl, 236: 27–36 12408501 10.1080/003655202320621427

[bib5] Ciceroni L, Ciarrocchi S (1998) Lyme disease in Italy, 1983–1996. New Microbiol 21: 407–418 9812324

[bib6] Ciceroni L, Ciarrochi S, Ciervo A, Mondarini V, Guzzo F, Caruso G, Murgia R, Cinco M (2001) Isolation and characterization of *Borrelia burgdorferi sensu lato* strains in an area of Italy where Lyme borreliosis is endemic. J Clin Microbiol 39: 2254–2260 11376066 10.1128/JCM.39.6.2254-2260.2001 PMC88120

[bib7] D'Elios MM, Amedei A, Del Prete G (2003) Helicobacter pylori antigen-specific T-cell responses at gastric level in chronic gastritis, peptic ulcer, gastric cancer and low-grade mucosa-associated lymphoid tissue (MALT) lymphoma. Microbes Infect 5: 723–730 12814773 10.1016/s1286-4579(03)00114-x

[bib8] de Koning J, Tazelaar DJ, Hoogkamp-Korstanje JA, Elema JD (1995) Acrodermatitis chronica atrophicans: a light and electron microscopic study. J Cutan Pathol 22: 23–32 7751475 10.1111/j.1600-0560.1995.tb00735.x

[bib9] Fierro MT, Novelli M, Savoia P, Cambieri I, Quaglino P, Osella-Abate S, Bernengo MG (2001) CD45RA+ immunophenotype in mycosis fungoides: clinical, histological and immunophenotypical features in 22 patients. J Cutan Pathol 28: 356–362 11437941 10.1034/j.1600-0560.2001.280704.x

[bib10] Forsberg P, Ernerudh J, Ekerfelt C, Roberg M, Vrethem M, Bergstrom S (1995) The outer surface proteins of Lyme disease borrelia spirochetes stimulate T cells to secrete interferon-gamma (IFN-gamma): diagnostic and pathogenic implications. Clin Exp Immunol 101: 453–460 7664493 10.1111/j.1365-2249.1995.tb03134.x PMC1553228

[bib11] Girschick HJ, Huppertz HI, Russmann H, Krenn V, Karch H (1996) Intracellular persistence of Borrelia burgdorferi in human synovial cells. Rheumatol Int 16: 125–132 8893378 10.1007/BF01409985

[bib12] Goodlad JR, Davidson MM, Hollowood K, Ling C, MacKenzie C, Christie I, Batstone PJ, Ho-Yen DO (2000) Primary cutaneous B-cell lymphoma and Borrelia burgdorferi infection in patients from the Highlands of Scotland. Am J Surg Pathol 24: 1279–1285 10976703 10.1097/00000478-200009000-00012

[bib13] Herne KL, Talpur R, Breuer-McHam J, Champlin R, Duvic M (2003) Cytomegalovirus seropositivity is significantly associated with mycosis fungoides and Sezary syndrome. Blood 101: 2132–2136 12446446 10.1182/blood-2002-07-2247

[bib14] Hofbauer GF, Kessler B, Kempf W, Nestle FO, Burg G, Dummer R (2001) Multilesional primary cutaneous diffuse large B-cell lymphoma responsive to antibiotic treatment. Dermatology 203: 168–170 11586019 10.1159/000051735

[bib15] Jackow CM, Cather JC, Hearne V, Asano AT, Musser JM, Duvic M (1997) Association of erythrodermic cutaneous T-cell lymphoma, superantigen-positive *Staphylococcus aureus*, and oligoclonal T-cell receptor V beta gene expansion. Blood 89: 32–40 8978274

[bib16] Jaenson TG (1991) The epidemiology of lyme borreliosis. Parasitol Today 7: 39–45 15463419 10.1016/0169-4758(91)90187-s

[bib17] Jaffe ES, Harris NL, Stein H, Vardiman JW (eds) (2001) World Health Organization Classification of Tumours: Pathology and Genetics of Tumours of Haematopoietic and Lymphoid Tissues. Lyon, France: IARC Press

[bib18] Kanegane H, Nomura K, Miyawaki T, Tosato G (2002) Biological aspects of Epstein–Barr virus (EBV)-infected lymphocytes in chronic active EBV infection and associated malignancies. Crit Rev Oncol Hematol 44: 239–249 12467964 10.1016/s1040-8428(02)00115-4

[bib19] Khan ZM, Sebenik M, Zucker-Franklin D (1996) Localization of human T-cell lymphotropic virus-1 tax proviral sequences in skin biopsies of patients with mycosis fungoides by *in situ* polymerase chain reaction. J Invest Dermatol 106: 667–672 8618002 10.1111/1523-1747.ep12345488

[bib20] Klemke CD, Dippel E, Dembinski A, Ponitz N, Assaf C, Hummel M, Stein H, Goerdt S (2002) Clonal T cell receptor gamma-chain gene rearrangement by PCR-based GeneScan analysis in the skin and blood of patients with parapsoriasis and early-stage mycosis fungoides. J Pathol 197: 348–354 12115881 10.1002/path.1133

[bib21] Kodama K, Massone C, Chott A, Metze D, Kerl H, Cerroni L (2005) Primary cutaneous large B-cell lymphomas: clinicopathologic features, classification, and prognostic factors in a large series of patients. Blood 106: 2491–2497 15947086 10.1182/blood-2005-03-1175

[bib22] Kraiczy P, Skerka C, Kirschfink M, Zipfel PF, Brade V (2001) Mechanism of complement resistance of pathogenic Borrelia burgdorferi isolates. Int Immunopharmacol 1: 393–401 11367524 10.1016/s1567-5769(00)00041-2

[bib23] Kraiczy P, Skerka C, Kirschfink M, Zipfel PF, Brade V (2002) Immune evasion of Borrelia burgdorferi: insufficient killing of the pathogens by complement and antibody. Int J Med Microbiol 291(Suppl 33): 141–146 12141738 10.1016/s1438-4221(02)80027-3

[bib24] Lebech AM (2002) Polymerase chain reaction in diagnosis of Borrelia burgdorferi infections and studies on taxonomic classification. APMIS Suppl, 105: 1–40 11985118

[bib25] Lehmann U, Kreipe H (2001) Real-time PCR analysis of DNA and RNA extracted from formalin-fixed and paraffin-embedded biopsies. Methods 25: 409–418 11846610 10.1006/meth.2001.1263

[bib26] Massone C, Kodama K, Kerl H, Cerroni L (2005) Histopathologic features of early (patch) lesions of mycosis fungoides: a morphologic study on 745 biopsy specimens from 427 patients. Am J Surg Pathol 29: 550–560 15767812 10.1097/01.pas.0000153121.57515.c6

[bib27] McCarthy KP, Sloane JP, Kabarowski JH, Matutes E, Wiedemann LM (1992) A simplified method of detection of clonal rearrangements of the T-cell receptor-gamma chain gene. Diagn Mol Pathol 1: 173–179 1342963

[bib28] Morales Suarez-Varela MM, Llopis Gonzalez A, Marquina Vila A, Bell J (2000) Mycosis fungoides: review of epidemiological observations. Dermatology 201: 21–28 10971054 10.1159/000018423

[bib29] Mullegger RR (2004) Dermatological manifestations of Lyme borreliosis. Eur J Dermatol 14: 296–309 15358567

[bib30] Nagasawa T, Takakuwa T, Takayama H, Dong Z, Miyagawa S, Itami S, Yoshikawa K, Aozasa K (2004) Fas gene mutations in mycosis fungoides: analysis of laser capture-microdissected specimens from cutaneous lesions. Oncology 67: 130–134 15539917 10.1159/000080999

[bib31] Nagore E, Ledesma E, Collado C, Oliver V, Perez-Perez A, Aliaga A (2000) Detection of Epstein–Barr virus and human herpesvirus 7 and 8 genomes in primary cutaneous T- and B-cell lymphomas. Br J Dermatol 143: 320–323 10951139 10.1046/j.1365-2133.2000.03657.x

[bib32] Oksi J, Savolainen J, Pene J, Bousquet J, Laippala P, Viljanen MK (1996) Decreased interleukin-4 and increased gamma interferon production by peripheral blood mononuclear cells of patients with Lyme borreliosis. Infect Immun 64: 3620–3623 8751908 10.1128/iai.64.9.3620-3623.1996 PMC174272

[bib33] Ornstein K, Berglund J, Nilsson I, Norrby R, Bergstrom S (2001) Characterization of Lyme borreliosis isolates from patients with erythema migrans and neuroborreliosis in Southern Sweden. J Clin Microbiol 39: 1294–1298 11283044 10.1128/JCM.39.4.1294-1298.2001 PMC87927

[bib34] Pachner AR, Dail D, Bai Y, Sondey M, Pak L, Narayan K, Cadavid D (2004) Genotype determines phenotype in experimental Lyme borreliosis. Ann Neurol 56: 361–370 15349863 10.1002/ana.20192

[bib35] Pancake BA, Zucker-Franklin D, Coutavas EE (1995) The cutaneous T cell lymphoma, mycosis fungoides, is a human T cell lymphotropic virus-associated disease. A study of 50 patients. J Clin Invest 95: 547–554 7860737 10.1172/JCI117697 PMC295510

[bib36] Pauluzzi P, Bonin S, Gonzalez Inchaurraga MA, Stanta G, Trevisan G (2004) Detection of spirochaetal DNA simultaneously in skin biopsies, peripheral blood and urine from patients with erythema migrans. Acta Derm Venereol 84: 106–110 15206688 10.1080/00015550310006815

[bib37] Perticarari S, Presani G, Prodan M, Granzotto M, Murgia R, Cinco M (2003) Lymphocyte apoptosis co-cultured with Borrelia burgdorferi. Microb Pathogenesis 35: 139–145 10.1016/s0882-4010(03)00096-2 12946326

[bib38] Ponti R, Quaglino P, Novelli M, Fierro MT, Comessatti A, Peroni A, Bonello L, Bernengo MG (2005) T-cell receptor gamma gene rearrangement by multiplex polymerase chain reaction/heteroduplex analysis in patients with cutaneous T-cell lymphoma (mycosis fungoides/Sezary syndrome) and benign inflammatory disease: correlation with clinical, histological and immunophenotypical findings. Br J Dermatol 153: 565–573 16120144 10.1111/j.1365-2133.2005.06649.x

[bib39] Rijpkema SG, Tazelaar DJ, Molkenboer MJ, Noordhoek GT, Plantinga G, Schouls LM, Schellekens JF (1997) Detection of Borrelia afzelii, *Borrelia burgdorferi sensu stricto*, *Borrelia garinii* and group VS116 by PCR in skin biopsies of patients with erythema migrans and acrodermatitis chronica atrophicans. Clin Microbiol Infect 3: 109–116 11864084 10.1111/j.1469-0691.1997.tb00259.x

[bib40] Robertson J, Murdoch S, Foster L, Green S (1999) Isolation and species typing of Lyme borreliosis spirochaetes from UK patients with erythema migrans. Eur J Epidemiol 15: 499–500 10442477 10.1023/a:1007591501411

[bib41] Ruzic-Sabljic E, Strle F, Cimperman J, Maraspin V, Lotric-Furlan S, Pleterski-Rigler D (2000) Characterisation of *Borrelia burgdorferi sensu lato* strains isolated from patients with skin manifestations of Lyme borreliosis residing in Slovenia. J Med Microbiol 49: 47–53 10628825 10.1099/0022-1317-49-1-47

[bib42] Saed G, Fivenson DP, Naidu Y, Nickoloff BJ (1994) Mycosis fungoides exhibits a Th1-type cell-mediated cytokine profile whereas Sezary syndrome expresses a Th2-type profile. J Invest Dermatol 103: 29–33 8027577 10.1111/1523-1747.ep12388985

[bib43] Sarris AH, Daliani D, Ulmer R, Crow M, Broxmeyer HE, Reiss M, Karasavvas N, Zelenetz AD, Pugh W, Cabanillas F, Deisseroth AB, Duvic M (1997) Interferon-inducible protein 10 as a possible factor in the pathogenesis of cutaneous T-cell lymphomas. Clin Cancer Res 3: 169–177 9815669

[bib44] Sarris AH, Esgleyes-Ribot T, Crow M, Broxmeyer HE, Karasavvas N, Pugh W, Grossman D, Deisseroth A, Duvic M (1995) Cytokine loops involving interferon-gamma and IP-10, a cytokine chemotactic for CD4+ lymphocytes: an explanation for the epidermotropism of cutaneous T-cell lymphoma? Blood 86: 651–658 7605995

[bib45] Scarpa C, Trevisan G, Stinco G (1994) Lyme borreliosis. Dermatol Clin 12: 669–685 7805296

[bib46] Schwaiger M, Peter O, Cassinotti P (2001) Routine diagnosis of *Borrelia burgdorferi* (*sensu lato*) infections using a real-time PCR assay. Clin Microbiol Infect 7: 461–469 11678928 10.1046/j.1198-743x.2001.00282.x

[bib47] Seiler KP, Weis JJ (1996) Immunity to Lyme disease: protection, pathology and persistence. Curr Opin Immunol 8: 503–509 8794009 10.1016/s0952-7915(96)80038-0

[bib48] Shimakage M, Sasagawa T, Kawahara K, Yutsudo M, Kusuoka H, Kozuka T (2001) Expression of Epstein–Barr virus in cutaneous T-cell lymphoma including mycosis fungoides. Int J Cancer 92: 226–231 11291050 10.1002/1097-0215(200102)9999:9999<::aid-ijc1172>3.0.co;2-o

[bib49] Shtrichman R, Samuel CE (2001) The role of gamma interferon in antimicrobial immunity. Curr Opin Microbiol 4: 251–259 11378475 10.1016/s1369-5274(00)00199-5

[bib50] Siegel RS, Pandolfino T, Guitart J, Rosen S, Kuzel TM (2000) Primary cutaneous T-cell lymphoma: review and current concepts. J Clin Oncol 18: 2908–2925 10920140 10.1200/JCO.2000.18.15.2908

[bib51] Silberer M, Koszik F, Stingl G, Aberer E (2000) Downregulation of class II molecules on epidermal Langerhans cells in Lyme borreliosis. Br J Dermatol 143: 786–794 11069457 10.1046/j.1365-2133.2000.03776.x

[bib52] Spellberg B, Edwards Jr JE (2001) Type 1/type 2 immunity in infectious diseases. Clin Infect Dis 32: 76–102 11118387 10.1086/317537

[bib53] Strle F (1999) Lyme borreliosis in Slovenia. Zentralbl Bakteriol 289: 643–652 10652723 10.1016/s0934-8840(99)80023-1

[bib54] Tan RS, Butterworth CM, McLaughlin H, Malka S, Samman PD (1974) Mycosis fungoides—a disease of antigen persistence. Br J Dermatol 91: 607–616 4281316 10.1111/j.1365-2133.1974.tb12449.x

[bib55] Tokura Y, Heald PW, Yan SL, Edelson RL (1992) Stimulation of cutaneous T-cell lymphoma cells with superantigenic staphylococcal toxins. J Invest Dermatol 98: 33–37 1728639 10.1111/1523-1747.ep12494184

[bib56] Van Krieken JH, Hoeve MA (2000) Epidemiological and prognostic aspects of gastric MALT-lymphoma. Recent Results Cancer Res 156: 3–8 10802857 10.1007/978-3-642-57054-4_1

[bib57] Weinstock MA, Horm JW (1988) Mycosis fungoides in the United States. Increasing incidence and descriptive epidemiology. JAMA 260: 42–46 3379722

[bib58] Widhe M, Jarefors S, Ekerfelt C, Vrethem M, Bergstrom S, Forsberg P, Ernerudh J (2004) Borrelia-specific interferon-gamma and interleukin-4 secretion in cerebrospinal fluid and blood during Lyme borreliosis in humans: association with clinical outcome. J Infect Dis 189: 1881–1891 15122525 10.1086/382893

[bib59] Wilske B (2003) Diagnosis of lyme borreliosis in europe. Vector Borne Zoonotic Dis 3: 215–227 14733674 10.1089/153036603322662200

[bib60] Wood GS, Salvekar A, Schaffer J, Crooks CF, Henghold W, Fivenson DP, Kim YH, Smoller BR (1996) Evidence against a role for human T-cell lymphotrophic virus type I (HTLV-I) in the pathogenesis of American cutaneous T-cell lymphoma. J Invest Dermatol 107: 301–307 8751960 10.1111/1523-1747.ep12363010

[bib61] Zanier L (1995–2000) Incidence of mycosis fungoides. Friuli-Venezia-Giulia cancer registry personal comunication incidence of mycosis fungoides from 1995 to 2000. In Friuli-Venezia-Giulia RT (ed). Agenzia Regionale della Sanità

[bib62] Zhang JR, Hardham JM, Barbour AG, Norris SJ (1997) Antigenic variation in Lyme disease borreliae by promiscuous recombination of VMP-like sequence cassettes. Cell 89: 275–285 9108482 10.1016/s0092-8674(00)80206-8

[bib63] Zucker-Franklin D, Pancake BA (1994) The role of human T-cell lymphotropic viruses (HTLV-I and II) in cutaneous T-cell lymphomas. Semin Dermatol 13: 160–165 7986683

